# Neuroinflammation: A Possible Link Between Chronic Vascular Disorders and Neurodegenerative Diseases

**DOI:** 10.3389/fnagi.2022.827263

**Published:** 2022-05-19

**Authors:** Emmanuel Moyse, Slavica Krantic, Nesrine Djellouli, Sébastien Roger, Denis Angoulvant, Camille Debacq, Victoire Leroy, Bertrand Fougere, Amal Aidoud

**Affiliations:** ^1^University of Tours, EA4245, Transplantation, Immunologie, Inflammation, Tours, France; ^2^Centre de Recherche Saint-Antoine (CRSA), Immune System and Neuroinflammation Laboratory, Hôpital Saint-Antoine, Inserm U938, Sorbonne Université, Paris, France; ^3^Department of Cardiology, Tours University Hospital, Tours, France; ^4^Division of Geriatric Medicine, Tours University Hospital, Tours, France; ^5^University of Tours, EA7505, Education, Ethics, Health, Tours, France

**Keywords:** inflammation, neuroinflammation, microglia, astrocytes, macrophages, ischemia-reperfusion, Alzheimer’s disease

## Abstract

Various age-related diseases involve systemic inflammation, i.e. a stereotyped series of acute immune system responses, and aging itself is commonly associated with low-grade inflammation or inflamm’aging. Neuroinflammation is defined as inflammation-like processes inside the central nervous system, which this review discusses as a possible link between cardiovascular disease-related chronic inflammation and neurodegenerative diseases. To this aim, neuroinflammation mechanisms are first summarized, encompassing the cellular effectors and the molecular mediators. A comparative survey of the best-known physiological contexts of neuroinflammation (neurodegenerative diseases and transient ischemia) reveals some common features such as microglia activation. The recently published transcriptomic characterizations of microglia have pointed a marker core signature among neurodegenerative diseases, but also unraveled the discrepancies with neuroinflammations related with acute diseases of vascular origin. We next review the links between systemic inflammation and neuroinflammation, beginning with molecular features of respective pro-inflammatory cells, i.e. macrophages and microglia. Finally, we point out a gap of knowledge concerning the atherosclerosis-related neuroinflammation, which is for the most surprising given that atherosclerosis is established as a major risk factor for neurodegenerative diseases.

## Introduction

Psychological disturbances are part of the “sickness syndrome” triggered by episodes of inflammation (Dantzer et al., 2008) and their occurrence has been correlated with increased morbidity in elderly people (Majnaric et al., 2021). Aging is commonly associated with increased inflammatory tone at the systemic level, which is summarized in the modern concept of inflamm’aging and is a risk factor for cognitive decline pathologies in aging (Fülop et al., 2018). Psychological frailty assessments must therefore include the inflammatory status of each subject.

Inflammation is defined as a stereotyped series of acute responses of the immune system in response to tissue injury or infection by pathogens, resulting in tissue repair and/or pathogen elimination (Okin and Medzhitov, 2012). Initial inflammatory response occurs in the context of innate immunity based on the detection of danger signal molecules (DAMP: damage-associated molecular pattern, and PAMP: pathogen-associated molecular pattern) by circulating or tissue-hosted sentinel cells (monocytes/macrophages, resident macrophages, mast cells, dendritic cells) *via* pattern recognition receptors (PRR). Activation of PRR yields production of microbe-destroying molecules such as reactive oxygen species (ROS) and reactive nitrogen species (RNS), phagocytosis of cell and tissue debris by macrophages and release of a plethora of immune cell-modulating chemical messengers (cytokines, chemokines, growth factors) (Takeuchi and Akira, 2010). Efficiency of the inflammatory response is due to its rapidity of setting but also on its transiency. A non-resolved acute inflammatory response can switch to a state of chronic inflammation, which involves recruitment of circulating leukocytes, notably B- and T- lymphocytes *via* the activation of the adaptive immune system (Chovatiya and Medzhitov, 2014). There is growing evidence that chronic systemic inflammation, even at low grade, is a detrimental process and a risk factor for many chronic diseases such as neurodegenerative and cardiovascular diseases (Ferrucci and Fabbri, 2018).

Neuroinflammation comprises inflammation-like processes inside the parenchyma of the central nervous system (CNS). It is currently well accepted that these inflammatory processes could take place locally within the CNS despite “the immune privilege of the brain,” i.e., absence of direct interaction of CNS parenchyma with systemic circulation-borne cells and soluble cell-communication messengers due to the blood-brain-barrier (BBB; DiSabato et al., 2016). Neuroinflammation is currently considered as a driving force in progression and likely etiology of numerous neurological diseases, including neurodegenerative ones. Some common mechanisms behind neuroinflammation characterizing a variety of neurological and neurodegenerative conditions are now emerging and may support novel therapeutic developments. However, such perspective is hampered by discrepant terminologies and experimental conditions used to study neuroinflammation. The present review aims to highlight (i) the common features of neuroinflammation beyond the diversity of physio-pathological models that have been addressed, (ii) the relationship between systemic inflammation and neuroinflammation, and (iii) the systemic inflammation and neuroinflammation cross-talk in the particular case of the age-related chronic cardio-vascular disorder, atherosclerosis.

## Neuroinflammation: The Innate Immune Reaction of the Central Nervous System

### Mechanisms and Effectors of Neuroinflammation

Innate immunity processes inside the CNS parenchyma occur in various neurological pathologies where they manifest by increased tissue levels of chemical messengers that are commonly correlated with peripheral inflammation (Layé et al., 1994; Dantzer, 1996; Quan et al., 1997). However, in the case of neuroinflammation, these chemical messengers are produced locally in the nervous parenchyma by specialized cells of the CNS (brain and spinal cord) such as glial cells and even by neurons. These chemical messengers participate to cell-to-cell communication and activation.

#### Neuroinflammation Typically Involves Four Categories of Stereotyped Mechanisms

(i) Increased tissue concentrations of chemical mediators like: cytotoxic molecules (ROS, produced along the mitochondrial respiratory chain, RNS including nitric oxide [NO], biosynthesis of which is catalyzed by inducible nitric oxide synthase [iNOS]; Chitnis and Weiner, 2017) prostaglandins, pro-inflammatory cytokines (e.g., TNF-α, IL-1β, IL6), anti-inflammatory cytokines (e.g., IL4, IL10, TGFβ), chemokines (CCL2, CCL5, CXCL1…), ATP and matrix metalloproteases (MMP2, MMP9…). The balance between production of pro- and anti-inflammatory cytokines depends upon pathological context and its time-course.
(ii) Functional activation and proliferation of microglia and astrocytes (the latter being the most abundant type of neural cells) in pathology-specific regions of the CNS (Radenovic et al., 2020).
(iii) Infiltration of peripheral immune cells from the systemic circulation: monocytes-macrophages, T-lymphocytes (including T-regulators; Dansokho et al., 2016) which is associated with permeabilization of the blood-brain-barrier (BBB) (Estes and McAllister, 2014).
(iv) Neuronal cell death, caused by the resulting neurotoxic context (Skaper et al., 2018).

**FIGURE 1:**
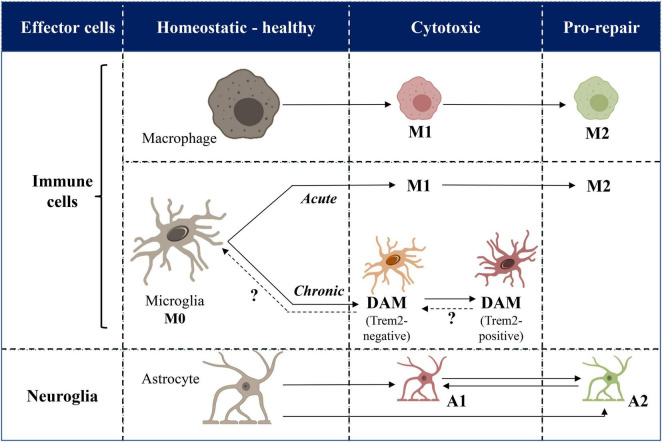
Cellular mechanisms of inflammation and neuroinflammation: a schematic picture. Immune and neural effector cells of inflammation and neuroinflammation are represented in horizontal rows. Healthy and pathological phenotypes are categorized in columns. Plain vs. dashed arrows indicate demonstrated vs. hypothetical switches from one phenotype to another. Acute vs. chronic diseases are indicated in italic. DAM, disease-associated microglia.

The involvement of glial cells in neuroinflammation deserves to be clarified in terms of clear distinction between glial cells of neural origin (i.e., astrocytes) and those related to immune system (i.e., microglia) to avoid confusion (Figure 1).

#### Microglia

Microglial cells play a central role in neuroinflammation by: i) sensing “danger signals” coming from pathogens or damaged “self” cells throughout the CNS parenchyma *via* their PRRs; ii) phagocytosis of tissue remnants issued from neural lesions; iii) production of chemical messengers and recruitment of other cellular effectors (DiSabato et al., 2016). Microglial cells belong to the hematopoietic lineage since they derive from monocyte/macrophage precursors that migrated from the yolk sac of early embryo to the neural tube before the establishment of the BBB (Prinz et al., 2021). Microglia is thus a non-neural glia: it shares many phenotypic and functional properties with peripheral macrophages but remains a distinct cell type (Prinz et al., 2021). Consistently, all microglia express constitutively the markers such as Iba1, CD11, HexB which are common with other cells of monocyte/macrophage lineage. In healthy adult CNS, microglial cells spread homogeneously throughout the parenchyma but display a panoply of both brain-region and sex-specific morphological features (Grabert et al., 2016). Microglial cells are characterized cytologically by a small size (cell body 15–30 mm diameter; Leyh et al., 2021), thin ramified expansions, expression of danger/damage sensors (scavenger receptors, DAMP and PAMP receptors, purinergic receptors P2Y12, P2×4, P2×7) and low phagocytic activity. These features globally qualify the “homeostatic phenotype” of microglia in healthy adult CNS (Butovsky and Weiner, 2018; Jiang and Roger, 2020; Masuda et al., 2020a).

#### Astrocytes

Neuroinflammation also involves activation of astrocytes, i.e., the predominant neural type of glia in the CNS, originating from the embryonic neurectoderm like neurons and myelin-elaborating glial cells (oligodendrocytes in the CNS and Schwan cells in the periphery). Astrocytes therefore cannot be designated as being “immune cells” even though they do participate in some immune processes; like in many other examples, the cell lineage must not be confounded with physiological function. It is, however, of utmost importance to recall that astrocytes and microglial cells are homogeneously intermingled throughout the tissue parenchyma of the CNS where they tightly cooperate functionally both in homeostatic and pathological conditions. Astrocytes are highly ramified cells that extend long and thin leaflets between neuronal processes and especially around synapses, which supports their well-known roles in regulation of neuronal activity and in metabolic support to neurons, extracellular fluid homeostasis and electrical isolation of excitable cells (Sofroniew, 2020). Their specific, genuine phenotypic marker is the glial fibrillary acidic protein (GFAP). Astrocytes express various DAMP and PAMP receptors, among which several Toll-like receptors (TLR) and purinergic receptors such as P2×7, which when activated by extracellular ATP, elicits neurodegeneration (Sidoryk-Wegrzynowicz and Struzinska, 2021; Territo and Zarrinmayeh, 2021; Zhao et al., 2021). Beyond relative cyto-morphological uniformity of astrocytes throughout the CNS, region-specific subpopulations are being unraveled with transcriptomic approaches (Khakh and Deneen, 2019; Diaz-Castro et al., 2021).

Remarkably, both microglia and astrocytes are characterized by their phenotypic plasticity which began to be uncovered in the last few years, particularly in pathological contexts of injuries or diseases. Such characterization has been long hampered because these two cell types are exquisitely sensitive to microenvironment and display phenotypic changes in primary cultures, which complicates the interpretation of *in vitro* studies in general, and characterization of their pathology-related plasticity, in particular.

### Pathological Contexts of Neuroinflammation: A Comparative Survey

Neuroinflammation has been reported as associated to acute lesions subsequent to trauma or to vascular obturation and/or rupture (causing, respectively ischemia and/or hemorrhage), and in relation with chronic neurodegenerative diseases (Alzheimer’s disease, Parkinson’s disease, Huntington’s, amyotrophic lateral sclerosis) and multiple sclerosis (Muzio et al., 2021). Neuroinflammation also occurs in relation with systemic inflammatory diseases as for instance type II diabetes (Moyse et al., 2019).

#### Differential Cellular and Molecular Mechanisms of Neuroinflammation

At the cellular level, microglia have been histologically characterized to undergo “activation” in the above pathological contexts, as early as by pioneering description by Del Rio Hortega one century ago (Leyh et al., 2021). Following either of above-mentioned pathological conditions, both in animal models and in post-mortem human brains, microglial cells in the affected area have been found to retract their ramifications toward a more roundish morphology, proliferate, acquire migration capacity by amoeboid motility, increase their phagocytic activity and switch their secretome to pro-inflammatory cytokines (IL1β, IL6, TNFα) and cytotoxic compounds (RNS, ROS) (Prinz et al., 2021; Wieronska et al., 2021). In parallel, astrocytes also proliferate and secrete matrix components which builds-up a “glial scar” around the affected area, and alter their secretome toward production of pro-inflammatory and chemotactic mediators (Giovannoni and Quintana, 2020). Astrocytes’ activation occurs in the anatomical vicinity of microglial activation in various neuropathological states *in vivo*, as analyzed by combined immunohistochemistry of their respective markers GFAP and Iba1 (Zamanian et al., 2012). Neuroanatomical location and time-course of these micro- and astro-glial changes nevertheless vary depending upon type of injury or disease (see below). Besides, it has to be stressed that other cell types are also involved in neuroinflammation, namely neurons through their constitutive communication with microglia *via* interaction of neuronal membrane-expressed fractalkine (CX3CL1) and its microglial receptor CX3CR1, but also endothelial cells and pericytes of CNS blood vessels (Estes and McAllister, 2014; DiSabato et al., 2016). Interestingly, endothelial cells use the neuroinflammation effector NO as a paracrine relay for physiological regulators of vasomotricity (Tousoulis et al., 2012).

At the molecular level, mechanisms of neuroinflammation have been unraveled in the last few years by *ex vivo* transcriptomic comparisons of microglial subpopulations (or single cells) dissociated after micro-dissection of the damaged brain regions in experimental animal models of disease or injuries at critical stages, as compared to analogous samples coming from the respective controls. Microglia was purified from primary cell suspensions by fluorescence-activated cell sorter (FACS) using for instance CD11b-fluorescent immunolabeling, and subjected to RNA-sequencing (Keren-Shaul et al., 2017; Krasemann et al., 2017; Madore et al., 2020; Masuda et al., 2020b; Li et al., 2021; Liu et al., 2021; Lu et al., 2021; Muzio et al., 2021; Yusuying et al., 2021). The key results of these approaches are summarized in Table 1 to facilitate comparison between the different pathological contexts of neuroinflammation. Overall, these data reveal both unity and diversity of mechanisms underlying neuroinflammation, as discussed below.

**TABLE 1:** Microglia phenotypes in health and disease.

Physio-pathology	Homeostatic microglia *DAMP sensing via PRR*	M1 phenotype *proinflammatory*	M2 phenotype *antiinflammatory*	DAM stage1	DAM stage2
				*pro-inflammatory*	*pro-inflammatory, neurotoxic*
Health	P2ry12, PY2ry13, P2X7R, Tmem119, SiglecH, Gpr34, Socs3, Olfml3, Fcrls, Cts3, Ctsd, Ctss, Sparc, C1qa, Tmsb4x, C1qb; **Sall1, Egr1** *Common with monocytes/macrophages*: CX3CR1, Fc, CD200R1, ITGAM (a-integrin=CD11b), CSF1R (=CD115), Iba1, ADGRE, SIRPA (=CD172a) ***IL10, TGFβ, (IL4)***				
Ischemia-reperfusion (mouse)		CD86, CD16/32, CD68, iNOS*^high^* ***IL1β, TNFα, IL6, NO, ROS***	CD206, Arg1, Ym1 iNOS*^low^* ***TGFβ, IL10, BDNF, VEGF, MMP-9***		
Alzheimer’s disease *Plaque-containing brain regions (tg mouse 5xFAD)*				**↘**CX3CR1, P2ry12, HexB Tmem119, TGFβR1, P2ry13, Gpr34, Olfm13; **Jun, Sall1, Egr1 ↗**ApoE, Tyrobp, Ctsb, Ctsd, B2m, Fth1, Lyz2 **↘*TGFβ***	**↗**Trem2, ApoE, Axl, Csf1, Clec7a, Lgals3, Itgax, Timp2, Cst7, Ctsl, Cd9, Ccl6, Ccl2, Lpl, Gpnmb, Ch25, Lilrb4, Spp1, Fabp5, P2X7R; **miR-155** **↗ *IL1β, TNFα, IL6, IL4, NO, ROS***
ALS *Spinal cord (tg mouse SOD1-/-)*				**↘** CX3CR1, P2ry12, Tmem119, Csf1r; **Olfml3, Sall1** **Up:** Trem2, ApoE, Axl, Clec7a, Itgax, Spp1; **miR-155** ***↘ TGFβ; ↗ IL1b, IL6, TNFα***	
Aging				**↘** P2ry12, P2ry13, SiglecH, Adora3 **↗** Trem2, ApoE, Tyrobp, Ctsd, Lpl **↘** ***TGFβ **↗** IL1b, IL6, TNFα, TGFβ1***	
Parkinson’s disease					
Pan-markers	Iba1, CD11b, HexB				

*Summarized from (i) Li et al., 2021; Liu et al., 2021; Lu et al., 2021; Yusuying et al., 2021 (for ischemia-reperfusion), (ii) Keren-Shaul et al., 2017; Krasemann et al., 2017; Butovsky and Weiner, 2018; Madore et al., 2020 (for Alzheimer’disease and amyotrophic lateral sclerosis), (iii) Muzio et al., 2021 (for aging); (iv) Madore et al., 2020; Li et al., 2021 (for Parkinson’s disease). Bold-italic: messengers secreted by microglial cells. Bold-underlined: genomic expression-controlling transcription factors and miRNA.*

Activated astrocytes could also be profiled by DNA-microarray or RNAseq on primary cells dissociated from rodent brain and purified by immunopanning (elimination of non-astrocyte cells by sequential incubations on plaques, respectively coated with antibodies directed against the cell-specific markers and/or astrocyte selection with antibody against a cell-surface marker like integrin subunit beta5 [Itbg5]). These approaches led to identify the genes that are upregulated in activated astrocytes from diverse neuroinflammation-linked pathologies in rodents, like the acute phase protein Lcn2 and the proteinase inhibitor SerpinA3N; however, most of astrocyte transcriptomic signatures were specific to the pathological context (ischemia and LPS exposure; Zamanian et al., 2012). Transcriptomic signatures of astrocytes purified *ex vivo* from animal models were defined as a proinflammatory “A1” phenotype in the context of acute CNS injury, LPS-induced neuroinflammation, neurodegenerative models and a pro-repair “A2” phenotype in the context of ischemia *in vivo* (Liddelow et al., 2017). However, further characterizations of purified astrocytes revealed a number of different pathology- and region- specific phenotypes that presently preclude generalization (Escartin et al., 2021).

#### Transient Vs. Chronic Neuroinflammatory States

In transient states of neuroinflammatory pathologies like ischemia-reperfusion (ischemia being triggered by 1-hour-long occlusion of the middle cerebral artery [MCAO] followed by reperfusion), CD11-positive microglia switches from the homeostatic phenotype (called M0) to a M1 phenotype characterized by pro-inflammatory properties, which converts 1 week later to a M2 resolutive phenotype with anti-inflammatory features and favoring tissue-repairing (Perego et al., 2011). Specific markers of M1 and M2 phenotypes have been characterized, in addition to the upregulation of genes encoding, respectively pro-inflammatory- (IL1β, TNFα, IL6) or anti-inflammatory (TGFb, IL10) cytokines (Table 1). Depending on *in vivo* context, M1 and M2 microglial phenotypes are mutually interconvertible, and each one can switch from M0 state. These microglial phenotypes are strikingly reminiscent of the M0, M1 and M2 phenotypes that had been characterized previously in peripheral monocytes/macrophages in the course of systemic inflammation (Table 2).

**TABLE 2:** Monocytes/macrophages phenotypes.

	Monocyte/peripheral macrophage M0	M1 macrophage *Pro-inflammatory; “classical” activation by IFNg or LPS*	M2 macrophage *anti-inflammatory, pro-repair; “alternative activation” by IL4*
Healthy adult mouse donor	*Common with homeostatic microglia*: Fc, ITGAM (a-integrin=CD11b), CD200R1, CSF1R (=CD115), Iba1, ADGRE, SIRPA (=CD172a), CX3CR1		
Adult mouse macrophages from bone marrow		Primary culture 1 week in GM-CSF and last 24h in LPS CD80, CD86, CD 40 ***IL1β, TNFα, IL6, NO, ROS, IL12, IL23, MMP1, MMP3, MMP13, ADAMTS***	Primary culture 1 week in M-CSF and last 24h in IL4 CD206, CD163, Arg1, PD-L2, RELMa ***TGFβ, IL10, IL1-RA, IGF, MMP-1, MMP12***

*Summarized from Shapouri-Moghaddam et al., 2018; Orecchioni et al., 2019; Spittau et al., 2020; Miyamoto et al., 2021. Bold-italic: messengers secreted by macrophages.*

In chronic states of neuroinflammation associated with neurodegenerative diseases (Alzheimer’s disease, amyotrophic lateral sclerosis [ALS]) and with aging, a pro-inflammatory phenotype of microglia has been characterized and designated “disease-associated microglia” (DAM). This phenotype shares some common features with the M1 phenotype above, in particular down-regulation of homeostatic microglia markers (the anti-inflammatory cytokine TGFb, the transcription factors Sall1 and Egr1). However, as far as available bibliography allows comparison, the down-regulation of microglial M0 markers is more extensive in the DAM phenotype than in the post-ischemic M1 above (Table 1). In addition, two sequential pro-inflammatory stages of DAM have been characterized in murine models of Alzheimer’s disease, according to the sequential up-regulation of the TREM2-ApoE pathway. Besides, ApoE, apart its function as the major apolipoprotein of the CNS interstitial fluid, is also expressed intracellularly in microglial cells and acts as a downstream mediator of the membrane receptor TREM2, to trigger further pro-inflammatory switch of microglia. The DAM “stage 2” (TREM2 + /ApoE +) is distinguished from “stage 1” (TREM2-/ApoE +) by upregulated expression of TREM2 and globally increased pro-inflammatory secretome and neurotoxicity (Keren-Shaul et al., 2017; Krasemann et al., 2017; Madore et al., 2020). The same approach on a murine transgenic model of another neurodegenerative disease (ALS) yielded a conserved core of transcriptomic signature in respective degenerating regions of the CNS, i.e.,: down-regulation of the M0-specific markers CX3CR1, P2Y12, Tmem119, Csf1r, Olfm13, Sall1; up-regulation of the DAM-specific markers Trem2, ApoE, Axl, Clec7a, Itgax, Spp1, miR-155; up-regulation of the pro-inflammatory secretome (IL1β, IL6, TNFα) (Table 1; Keren-Shaul et al., 2017; Krasemann et al., 2017) which led to postulate a “neurodegenerative signature” of microglia (Madore et al., 2020). It is striking to uncover such common features of microglia properties between two diseases with such different neurological outcomes and specific neuro-anatomic lesion (hippocampus and discrete neocortex areas in Alzheimer’s disease vs. ventral horn of spinal cord in ALS) (Keren-Shaul et al., 2017; Krasemann et al., 2017; Madore et al., 2020). However, to fully support the postulated generalization, the “neurodegenerative microglial signature” should now be assessed in the respective lesioned areas of other neurodegenerative diseases: substantia nigra and striatum in Parkinson’s disease, striatum in Huntington’s disease, hippocampus and lesioned areas of cerebral neocortex in Creutzfeld-Jacob disease. On the basis of already published studies, this aim could be performed merely by RT-qPCR of DAM vs. M0 markers on RNA extracts from micro-dissected explants of the respective lesioned area in murine models of these diseases.

Nevertheless, it has to be stressed that DAM phenotype, although displaying some dissimilarities with the M1 phenotype (e.g., ApoE is down-regulated in M1 but up-regulated in DAM), also presents some analogies with the M2 phenotype (e.g., up-regulated Arg-1 expression found in both DAM and M2), (Krasemann et al., 2017). By contrast, the relationship between the M1-M2 and putative DAM microglial phenotypes in models of acute, transient neuroinflammation (e.g., ischemia/reperfusion, see above), has not been assessed so far.

Interestingly, the transcriptomic approach on *ex vivo* brain tissue from a murine model of Alzheimer’s disease recently led to characterization of a novel phenotype of activated microglia, distinct from Trem2-ApoE-Clec7a-expressing DAM, but still increasing (as DAM) along disease progression. This new sub-population of microglia has been designated as “interferon response microglia” (IRM), and (as DAM) was identified in the vulnerable brain areas (hippocampus and cortex in the studied model of Alzheimer’s disease). The IRM upregulate a subset of genes characteristic for monocytes/microglia response to interferon-γ exposure (C1qa, C1qb, C1qc, Ctsb, Ctsd, Fth1, Lyz2) (Sala Frigerio et al., 2019). By analogy to interferon-γ (IFNγ) - dependent phenotypic switch in monocyte-macrophage lineage classically observed *in vitro*, and in the light of reported IFNγ increase the hippocampus after global transient ischemia in a rat model (Yasuda et al., 2011), an exciting hypothesis to test in the future studies is whether IRM might be induced in models of acute, transient neuroinflammation associated with ischemia/reperfusion.

Altogether, neuroinflammation thus displays both common and distinct features in different physio-pathological contexts (Figure 1). On the basis of the molecular profiling of microglia that has been recently achieved in a limited number of animal models, the translation of knowledge is now needed to fill the gaps in additional pathological models of neurological and neurodegenerative diseases associated with neuroinflammation in the chronical context. Furthermore, the transcriptomic assessment of neuroinflammatory profile of microglia in the acute settings such as ischemia/reperfusion models, never performed before, is also urgently warranted.

## Relationship Between Systemic Inflammation and Neuroinflammation

During the last two decades, systemic inflammation has been extensively documented to trigger neuroinflammation as an indirect consequence of inflammation signaling to the CNS parenchyma. Four major routes have been identified for such signaling (Dantzer et al., 2008; Barbosa-Silva et al., 2021):

– Activation of autonomic afferent nerves (vagal and trigeminal cranial ones) by the circulating pro-inflammatory cytokine IL1β, *via* its specific receptors at the surface of sensory fibers; in line, peripheral inflammation-induced sickness behavior is blocked by experimental vagotomy in rodents (Bluthé et al., 1996);
– Neurohumoral activation of PAMP- and DAMP- receptors of macrophages and resulting secretion of pro-inflammatory mediators in the BBB-devoid circumventricular organs and choroid plexus (Konsman et al., 2004);
– “IL-1β pathway” *via* specific receptor-mediated uptake of some cytokines in the BBB of the CNS microvasculature and activation of its endothelium (Erickson and Banks, 2018);
– Blood-brain-barrier disruption, which allows systemic pro-inflammatory mediators and immune cells to infiltrate the CNS parenchyma (Banks and Erickson, 2010).

In addition, the microvasculature in meninges and choroid plexus harbors a variety of myeloid cells: monocytes, macrophages, dendritic cells, granulocytes. However, the contribution of these cells to neuroinflammation *via* cross-talk with systemic inflammation is presently still controversial (reviewed in Herz et al., 2021).

Nevertheless, the inter-play between systemic and CNS inflammation is increasingly recognized. One of the broadly studied examples of such cross-talk is sepsis. Indeed, sepsis and, more generally, various pathogenic infections, yielding a “cytokine storm,” were documented to trigger encephalopathies and neuroinflammation which can be alleviated by peripheral anti-inflammatory therapies (Barbosa-Silva et al., 2021).

Besides, peripheral inflammation has been established as a risk factor for neurodegenerative diseases, especially Alzheimer’s disease. Of note, neurodegenerative lesions and cognitive deficits in this disease are preceded by neuroinflammation in animal models (Moyse et al., 2019). Moreover, at least in murine models of Alzheimer’s disease, recent studies demonstrated that both neuropathological lesions and cognitive deficits can be prevented or delayed by peripheral anti-inflammatory treatments (Bettcher et al., 2021).

Concerning acute injury settings, in a recent clinical trial, neuroinflammatory response to traumatic brain injury (TBI) has been alleviated by a peripheral treatment with an IL1β receptor antagonist, anakinra (Kineret), while peripheral inflammation markers remained unaffected (Lassaren et al., 2021). This clinical study indicates the causal role of systemic mediators in modulating TBI-linked neuroinflammation.

In spite of the great achievements accomplished during the last decades in terms of understanding the inter-play between systemic- and central (neuro)-inflammation, the biggest challenge for the future remains to decipher the underlying mechanisms and uncover putative common effectors. In this light, the purinergic P2×7 receptor, has recently attracted much interest since it has been causally involved in inflammatory changes, as well as in some psychiatric diseases like depression, both in human (Roger et al., 2010; Meyer et al., 2020) and in murine models (Chen et al., 2017; Bhattacharya and Jones, 2018; Martinez-Frailes et al., 2019; Troubat et al., 2021).

## The Case of Chronic Vascular Diseases

Atherosclerosis is a chronic disease characterized by progressive development of lipid-rich fibrotic deposits (atheroma plaques) inside the intima of large- and medium-sized arteries (Taleb, 2016). Increasing evidence points to the chronic inflammation, occurring either locally or at the systemic level, as a key factor in progression of atherosclerotic lesions and related acute cardiovascular events. Relevantly, various serum biomarkers of inflammation have been proposed as predictors of cardiovascular complications both in patients suffering from chronic vascular diseases (CVD) and in healthy adults, independently of CVD risk factors (Moriya, 2019). Notably, inflammatory biomarkers such as C-reactive protein (CRP) and interleukins (IL) -1 and -6 have been widely explored as biomarkers of endothelial dysfunction and inflammation in clinical studies (Libby, 2021a). The serum CRP, although relatively low in atherosclerotic patients, has been reported to be on average above the gauge of systemic inflammation (plasma level of 2 mg/L). Furthermore, IL-1-neutralizing treatment could reduce serum CRP levels and the risk of cardiovascular death (Libby, 2021b). Combined, these recent data suggest that atherosclerosis should be considered as a chronic inflammatory disease.

Given the growing evidence pointing to the impact of systemic inflammation as a trigger of neuroinflammation, and the fact that neuroinflammation is recognized as being associated with neurodegenerative diseases such as Alzheimer’s disease, it is important to assess neuroinflammation in the specific context of atherosclerosis in future studies.

Indeed, the mechanistic link between atherosclerosis and neuroinflammation has barely been addressed so far, except in one experimental study on the animal model of atherosclerosis: the ApoE-knockout (ApoE-/-) adult mouse fed for 2 months with a hyperlipidic diet (Denes et al., 2012). In the brain of this atherosclerosis mouse model, Iba1-immunoreactive microglial cells and CD45-immunopositive infiltrated leukocytes significantly outnumbered microglia and leukocytes seen in age-matched, wildtype mice (Denes et al., 2012). Moreover, *in vivo* IL-1β neutralization in ApoE-/- mice exposed to an atherogenic diet (either by administration of an IL-1β antibody, or by genetic crossing with IL1R1-/- mouse) could block neuroinflammatory features (VCAM-labeled vascular inflammation, increased number of Iba1-immunoreactive activated microglial cells, decreased intracerebral accumulation of CD45 + leukocytes in the brain) and reduced the atheroma burden in large arteries (Denes et al., 2012). Besides, the latter study explicitly demonstrated that IL-1β is causally related to atherosclerosis-associated neuroinflammation, and that systemic inflammation and neuroinflammation are inter-related. However, these data need to be extended by detailed characterization of microglia and astrocytes transcriptomic signatures in this model of atherosclerosis.

Epidemiological, genetic and clinical data indicate a consistent association between CVD and dementia. Alzheimer’s disease, the major cause of dementia world-wide, and CVD affect the same population of patients that shares many common risk factors. Accordingly, two recent autopsy studies showed an increased prevalence of CVD (Toledo et al., 2013) and atherosclerotic lesions (Arvanitakis et al., 2016) in Alzheimer’s disease patients, as compared to healthy controls. A correlation was also established between Alzheimer’s disease progression and the severity of atherosclerotic lesions, suggesting that CVD could be considered as a risk factor for development of Alzheimer’s disease. Moreover, cardioprotective treatments, such as angiotensin receptor antagonists and diuretics, yielded significantly reduced amyloid-β accumulation (the latter is a key histopathological feature of Alzheimer’s disease) (Glodzik et al., 2016). In addition, allele ε4 of the apolipoprotein E gene (*APOE*) is a risk factor for both Alzheimer’s disease (Wang et al., 2014) and CVD (Schächter et al., 1994). There is also a significant overlap between the mechanisms involved in Alzheimer’s disease and CVD. Indeed, decreased cerebral blood flow, morphological changes in the vascular system similar to those observed during arterial aging, permeability of the blood-brain barrier and cholinergic neurodegeneration are all found in both pathologies (Santos et al., 2017). Of note, systemic hypertension that is also linked to cognitive impairment, has been suggested to be both a trigger and consequence of neuroinflammation through activation of the renin-angiotensin system (Winklewski et al., 2015). These pathological processes could be synergistic, so that the pathological alterations of one accelerates the progression of the other. This view remains controversial and clinical data suggest rather additive effects.

More studies are clearly needed to further assess the putative analogy between CVDs and Alzheimer’s disease, notably regarding neuroinflammatory changes and though beyond chronic, progressive and age-related character of both pathologies. Indeed, neuroinflammation has been extensively characterized in the context of Alzheimer’s disease, especially by using transgenic murine models, but much less is known in relation with chronic CVDs like atherosclerosis. Neuroinflammation therefore deserves extensive characterization in the context of atherosclerosis. These future studies will help answering whether CVD-associated neuroinflammation displays a chronic feature and is closer to the neuroinflammation seen in Alzheimer’s disease rather than acute, transient context of ischemia-reperfusion.

## Conclusion

Neuroinflammation has been discovered some 30 years ago and first described as innate immunity response triggered inside the CNS parenchyma by systemic infection or CNS injury. More recently, data coming from a variety of pathological and physiological contexts in mammals, including human, has unraveled neuroinflammation as a pathophysiological process displaying many similarities with peripheral inflammation. Among these similarities, there is for instance the phagocytosis by circulating- or brain resident macrophages and use of common chemical messengers (e.g., cytokines and chemokines) for intercellular communication. Neuroinflammation, however, displays many specificities, related for example to the anatomical structure of the brain. Thus, specialized effector cells that are intermingled inside the brain parenchyma, are locally conditioned by their mutual interaction. These effector cells include: (i) microglia, i.e., resident macrophages deriving from non-neural hematopoietic lineage and thus belonging thus to the “immune cells” of the organism, and (ii) astrocytes from neuroectodermal (neural) lineage. Each of these cell types can undergo context-dependent switch between different phenotypes, from “homeostatic” states in physiological conditions to “disease-associated” ones in pathological contexts. These switches can be reversible in acute aggressions or definitive in chronic diseases like neurodegenerations. Molecular characterization of these phenotypes in animal models of a few pathologies, especially ischemia paradigms and Alzheimer’s disease, has revealed respective transcriptomic signatures that are different between acute and chronic diseases (Figure 1). In this light, the mechanisms behind the atherosclerosis-related neuroinflammation still remain poorly understood, since the phenotypes of effector cells and the transcriptomic variations of inflammatory mediators have not been addressed so far. The only exception is a single study published a decade ago (Denes et al., 2012), before discovery of the transcriptomic diversity of glial cells. It is urgent to fill this gap, since atherosclerosis is increasingly recognized as a major risk factor for neurological and neurodegenerative diseases which are ever-increasing in the aging populations world-wide.

## List of Transcriptomic Markers Cited

**ADGRE:** Adhesion G protein coupled receptors, i.e., a family of G protein-coupled receptor with seven transmembrane domains and an extracellular domain containing repeated Epidermal Growth Factor (EGF)-like calcium binding domains. ADGRE1 is a monocyte-macrophage marker (Waddell et al., 2018).

**ApoE:** Apolipoprotein E, i.e., the predominant member in central nervous system of the apolipoprotein family. These amphiphilic proteins bind cholesterol and transport it in extracellular fluids as lipoproteic particles. ApoE in addition, can be expressed inside activated microglial cells and transduces TREM2-detected signals (Krasemann et al., 2017). ApoE occurs under several isoforms, the e4 of which is a strong genetic risk factor of Alzheimer’s disease.

**Arg1:** Arginase-1.

**Axl:** A receptor tyrosine kinase (RTK) that specifically binds Growth-Arrest-Specific protein 6 (Gas6) and is expressed on microglia. Axl signaling reduces expression of the pro-inflammatory cytokine TNFα and thus dampens immune-mediated insult to the central nervous system (Weinger et al., 2011). Axl belongs to the TAM sub-family of RTKs along with Tyro3 and Mer, defined by pivotal roles in innate immunity (Lemke and Rothlin, 2008; Fourgeaud et al., 2016).

**C1q (a,b,c):** the initial, antigen-binding element of the “complement cascade” that triggers clearing of microbes or damaged endogenous cells in the frame of innate immune reaction.

**CD:** Cluster of Differentiation, followed by a number between 1 and 371, i.e., a cell surface glycoprotein used for immunophenotyping of immune cells.

**Clec7a:** C-type Lectin domain containing protein family 7 member A (or Dectin-1).

**Csf1r:** Colony stimulating factor-1 receptor, i.e., the transmembrane tyrosine kinase receptor for the macrophage growth factor CSF-1 (colony stimulating factor-1).

**Cts (b, d, s, 3):** Cathepsines, i.e., lysosomal proteases.

**CX3CL1:** Fractalkine, a member of the CX3C chemokine family. It is constitutively expressed by neurons and can be induced in astrocytes, microglia and endothelium (Pawelec et al., 2020). Fractalkine exists as either a membrane-integrated glycoprotein or a soluble, metalloprotease-cleaved isoform which, respectively mediate cell adhesion or chemotaxis.

**CX3CR1:** the fractalkine receptor, exclusively expressed by microglia (Pawelec et al., 2020).

**Egr1:** a transcription factor driving coordinate expression of diverse genes altogether defining the homeostatic M0 phenotype of microglia.

**Fc:** crystallizable fragment of antibody molecules, i.e., the immunoglobulin region that binds immune cells’activating receptor-Fc when complexed with antigen.

**Fcrls:** Fc receptor-like S, a transmembrane scavenger receptor.

**Fth1:** Ferritin Heavy chain-1

**Gpr34:** A G protein-coupled transmembrane receptor, once orphan, now related with the P2Y family of purinergic receptors (Schönberg et al., 2018). Gpr34 binding maintains microglia in its homeostatic (M0) state.

**HexB:** Hexosaminidase-B, i.e., a subunit of a lysosomal enzyme in microglia (Masuda et al., 2020a).

**Iba1:** Ionized calcium-binding adaptor molecule 1, a pan-marker of both microglia and systemic macrophages.

**iNOS:** inducible nitric oxide synthase (Sierra et al., 2014).

**Itgam ( = CD11b):** Integrin Subunit Alpha M, i.e., an heterodimeric transmembrane protein that mediates the phagocytosis of complement-coated particles by macrophages and combines with Itgax in mediating adherence of monocytes and neutrophils to activated endothelial cells.

**Itgax ( = CD11c):** Integrin Subunit Alpha X, i.e., an heterodimeric transmembrane protein combining with Itgam in mediating adherence of monocytes and neutrophils to activated endothelial cells.

**Lgals3:** Galectine-3, i.e., a ubiquitous immunoglobulin-E with glycan-binding property in both intra- and extra-cellular compartments, which modulates proliferation, cell death and survival, cell adhesion and cell migration (Ramirez et al., 2019). Lgals3 is expressed by both astrocytes and microglia, and its genetic deletion in mouse protects brain against injury. Lgals3 is a ligand of TREM2.

**Lyz2:** a lysosomal precursor (lysozyme) for a transglycolase-hydrolase enzyme of monocytes-macrophages, that targets peptidoglycans and chitin-oligosaccharides of bacterial walls.

**miR:** microRNAs are non-coding, short (around 20-nucleotide-long) RNAs that regulate expression of target proteins by specific hybridization to their mRNAs.

**NO:** nitric oxide, i.e., a gaseous liposoluble messenger with a very short half-life, that elicits its biological actions *via* direct binding to intracellular, second messenger-producing guanylate cyclase enzyme without specific receptor. NO can elicit toxicity on cells indirectly *via* its oxidized metabolites.

**Olfm13:** a member of the olfactomedins family, i.e., glycoproteins sharing an adhesive protein-protein domain, the prototype of which was discovered in olfactory epithelium and later expanded into diversified regulators of neural and immune development and plasticity (Anholt, 2014). Olfactomedin-13 is expressed in microglia but not in monocytes-macrophages.

**P2Y12, P2Y13:** G protein-coupled metabotropic ATP receptors.

**P2×4, P2×7:** Ionotropic ATP-gated receptors.

**Sall1:** a transcription factor driving expression of diverse genes defining the homeostatic M0 phenotype of microglia.

**Siglec:** Sialic acid binding immunoglobulin-like lectin, i.e., a family of receptors (16 members) recognizing the sialic groups in tissue glycocalyx structures. Siglec receptors are expressed specifically in immune cells, where they transduce inhibitory signals (Siddiqui et al., 2019).

**Socs3:** Suppressor Of Cytokine Signaling-3, i.e., a retro-inhibitor of ligand-bound cytokine receptors, promoting acute arrest of the cytokine message and long-term desensitization of cytokine receptors.

**Sparc:** Secreted protein acidic and rich in cysteine, i.e., a cell-matrix modulating protein involved in blood-brain-barrier function and in angiogenesis.

**Spp1:** osteopontine, i.e., an adhesion protein of bone tissue being expressed by bone resident macrophages (osteoblasts) and by microglia.

**Tmem119:** A transmembrane protein specifically expressed by microglia, to the exclusion of other immune cells and of neural cells (Bennett et al., 2016).

**Tmsb4x:** Thymosin beta 4 X-linked, i.e., an actin sequestering protein involved in regulation of actin polymerization and, as such, in cell division, migration and differentiation.

**TREM2:** Triggering Receptor Expressed on Myeloid cells-2, a phagocytic receptor expressed specifically in cells of the myeloid hematopoietic lineage (monocytes-macrophages, osteoclasts, dendritic cells and microglia). TREM2 senses phospholipids, apoptotic cells and lipoproteins. TREM2 mutations are risk factor for sporadic Alzheimer’s disease, and transgenic TREM2 deficiency in an AD murine model accelerates amyloid plaque deposition and neuron loss (Colonna and Wang, 2016; Herz et al., 2021). Mutation in TREM2 gene is a strong risk factor of Alzheimer’s disease.

**Tyrobp:** Tyro protein tyrosine kinase binding protein, forming a signaling complex with TREM2.

**Ym1:** Chitinase-3-like protein, i.e., a member of the glycosyl-hydrolase family that is activated during injury and inflammation, inhibits apoptosis and favors tissue repair and protection along with anti-inflammatory effects (Lee et al., 2011).

## Author Contributions

EM: cellular and molecular neurobiology and first draft writing. SK: neuroinflammation in Alzheimer’s disease. ND: transcriptomic assays of microglia in an animal model of neuroinflammation. SR: molecular effectors of inflammation in ischemia/reperfusion. DA: cardiovascular pathophysiology and inflammation. CD: neurocognitive pathologies in clinics. VL: neurocognitive pathologies and education and ethics in health. BF: aging-related frailty and systemic inflammation. AA: atherosclerosis, inflammation, and vaccination. All authors contributed to the article and approved the submitted version.

## Conflict of Interest

The authors declare that the research was conducted in the absence of any commercial or financial relationships that could be construed as a potential conflict of interest.

## Publisher’s Note

All claims expressed in this article are solely those of the authors and do not necessarily represent those of their affiliated organizations, or those of the publisher, the editors and the reviewers. Any product that may be evaluated in this article, or claim that may be made by its manufacturer, is not guaranteed or endorsed by the publisher.
